# Biomimetic extracellular matrix mediated somatic stem cell differentiation: applications in dental pulp tissue regeneration

**DOI:** 10.3389/fphys.2015.00118

**Published:** 2015-04-21

**Authors:** Sriram Ravindran, Anne George

**Affiliations:** Department of Oral Biology, University of Illinois at ChicagoChicago, IL, USA

**Keywords:** biomimetics, extracellular matrix, collagen, chitosan, dental pulp regeneration, dental pulp stem cells, bone marrow stem cells

## Abstract

Dental caries is one of the most widely prevalent infectious diseases in the world. It affects more than half of the world's population. The current treatment for necrotic dental pulp tissue arising from dental caries is root canal therapy. This treatment results in loss of tooth sensitivity and vitality making it prone for secondary infections. Over the past decade, several tissue-engineering approaches have attempted regeneration of the dental pulp tissue. Although several studies have highlighted the potential of dental stem cells, none have transitioned into a clinical setting owing to limited availability of dental stem cells and the need for growth factor delivery systems. Our strategy is to utilize the intact ECM of pulp cells to drive lineage specific differentiation of bone marrow derived mesenchymal stem cells. From a clinical perspective, pulp ECM scaffolds can be generated using cell lines and patient specific somatic stem cells can be used for regeneration. Our published results have shown the feasibility of using pulp ECM scaffolds for odontogenic differentiation of non-dental mesenchymal cells. This focused review discusses the issues surrounding dental pulp tissue regeneration and the potential of our strategy to overcome these issues.

## Introduction

Tissue engineering of dental and craniofacial tissues has gained enormous prominence in the past decade. In spite of these advances, clinical translation has remained elusive. One of the primary reasons lies in the specialized nature of dental tissue cell types and the limited availability of somatic stem cells that can be used to regenerate these tissues.

As proposed more than two decades ago by Langer and Vacanti, tissue engineering requires a triad of cells, scaffolds and growth factors (Langer and Vacanti, 1993). However, the approach toward maximizing the potential of this triad has been evolving consistently. In this focused review, we will discuss our biomimetic approach aimed at regeneration of dental pulp tissue. Additionally, we will also discuss the potential of this technology for regeneration of other tissues.

## Dental caries and pulp tissue

Dental caries is one of the most prevalent infectious disease in the world second only to the common cold (Islam et al., 2007). In the United States, it is the most widely prevalent infectious disease. A world health organization (WHO) report states that approximately 90% of the world's population has experienced dental caries. Poor oral hygiene is the primary cause for caries. However, several medical treatments such as radiation therapy and chemotherapy can also cause caries. Additionally, the use of drugs that negatively affect salivation can also result in caries. The disease is characterized by bacterial infection of the teeth. The pathogenic bacteria generate an acidic environment that slowly degrades the enamel and dentin hydroxyapatite and eventually exposes the dental pulp tissue for infection. Dental caries in its advanced stage is characterized by infected necrotic dental pulp tissue.

The dental pulp tissue is a highly vascularized and innervated specialized soft tissue that provides vitality to the tooth. The functions of dental pulp tissue range from response to bacterial insult and injury to transmission of mechanical stimuli and promotion of regeneration. However, the tissue itself possesses limited regenerative potential due to its anatomical arrangement and the specialized nature of dental pulp and odontoblast cells.

KEY CONCEPT 1 **Dental pulp and caries**

Dental pulp is a highly vascularized and innervated soft tissue that fills the root canal space. This soft tissue provides vitality to the tooth by serving as a mechano transducer of physical stimulus, by providing regenerative ability to form reparative dentin resulting from minor insult or injury and also by providing immediate response to bacterial insult. Dental caries is caused by bacterial infection of the dental pulp tissue leading to necrotic pulp tissue. It occurs primarily due to lack of oral hygiene, but can also be a result of several medical treatments such as chemotherapy.

## Root canal therapy

The current treatment for infected necrotic pulp is root canal therapy. **Root canal therapy** involves removal of the infected pulp tissue, disinfection of the pulp chamber, and filling of the chamber with gutta percha, a synthetic compound devoid of ability to support or promote regeneration of living tissue. As a result, the tooth loses its sensitivity and vitality and becomes a dead tooth. Therefore, teeth subjected to root canal therapy cannot respond immunologically to subsequent infections. Although the root canal space is disinfected prior to therapy, as a result of immunological inertness, subsequent secondary infections arising due to lack of oral hygiene or other continuing causes may go unnoticed until they spread to neighboring tissues resulting in a chronic condition that significantly affects the quality of life. In some rare cases, this might lead to loss of life as a result of sepsis.

KEY CONCEPT 2 **Root canal therapy**

This is the current treatment for necrotic pulp tissue. This treatment is characterized by removal of the necrotic pulp tissue, disinfection of the root canal space and filling up the pulp chamber with an inorganic compound followed by capping.

In children and adolescents with immature permanent teeth, root canal therapy poses an even greater problem by preventing root maturation. The result is loos of tooth due to cervical root fracture. This type of failure is the leading cause for treatment failure in immature teeth (Cvek, 1992). In order to preserve the vitality of the tooth in adults and to promote normal root maturation and longevity of immature permanent teeth in children, a regenerative strategy is required as a replacement for root canal therapy.

## Current approaches to dental pulp tissue regeneration

The drawbacks of root canal therapy in adults and children can be overcome if regeneration of functional pulp tissue can be accomplished clinically. An appropriate stem cell source is required to accomplish this task successfully. Dental pulp stem cells (DPSCs) are currently the preferred cell type for dental pulp tissue regeneration. DPSCs are mesenchymal stem cells that reside within the pulp tissue and are capable of multi-lineage differentiation (Shi et al., 2001; Shi and Gronthos, 2003; Kerkis et al., 2006; Huang et al., 2009). Other stem cell sources for pulp tissue engineering include stem cells from exfoliated deciduous teeth (SHED), stem cells from apical papilla (SCAP), and periodontal ligament derived stem cells (PDLSCs).

Dental pulp tissue engineering has been investigated *in vivo* in small animal models using DPSCs (Gronthos et al., 2002; Demarco et al., 2010; Karaoz et al., 2011), SHED (Cordeiro et al., 2008; Rosa et al., 2013), SCAP (Huang et al., 2010), and PDLSCs (Ravindran et al., 2014a, 2012b) and a variety of growth factors that includes fibroblast growth factors (FGFs) (Morito et al., 2009) and bone morphogenetic proteins (BMPs) (Nakashima, 1994; Nakashima and Reddi, 2003).

Recently, dental pulp tissue engineering was attempted in a canine pulpectomy model in mature (Iohara et al., 2011) and immature teeth (Wang et al., 2013). The studies were performed using a side population of DPSCs (CD105 +ve cells) and stromal derived factor 1 (SDF1) was used as the growth factor of choice to induce functional pulp tissue regeneration including vascularization and innervation. Additionally, DPSCs when used in conjunction with granulocyte stimulating factor (GSF) to isolate a side population of cells exhibiting higher mobility, showed an improved regenerative potential. Additionally, no change was observed in regenerative potential with cells isolated from aged dogs or regeneration in aged dogs in general when this combination was used (Iohara et al., 2013; Nakashima and Iohara, 2014). However, the caveat, as acknowledged by the authors, is that the clinical translation of the research is limited by the availability of viable autologous dental pulps.

Additionally, DPSCs have also been used to regenerate bone. Recent studies in human graft models show the potential of autologous DPSCs to regenerate bone in a clinical setting. This study provides a valuable insight into the use of DPSCs clinically (D'aquino et al., 2009).

## Limitations of dental stem cells

Dental stem cells are the ideal cell types for regeneration of dental tissues. However, the primary drawback associated with all of the dental stem cells mentioned above is their limited availability (Feng and Lengner, 2013). The easiest and most accessible way of isolating DPSCs is from the extracted third molars of patients (Tirino et al., 2012). However, in many cases, the third molars are infected or removed as a result of caries. Additionally, the number of cells obtained will not be sufficient to treat multiple carious teeth or to treat any teeth that may be infected in the future. On the other hand, DPSCs are also available for isolation from the inflamed pulp tissues (Alongi et al., 2010). These cells possess similar potential compared to DPSCs from natural pulp, albeit slightly altered stem cell characteristics. If successful, this technique can prove to be quite valuable. However, the success depends on the quality of the inflamed pulp and also the amount of cells that can be isolated from it.

SHED are stem cells that need to be isolated from deciduous teeth. However, their effectiveness in clinical therapy is heavily dependent upon development of reliable stem cell banking technologies. Even if such technologies are developed the procedure may not be cost effective for most of the population as a viable option for treatment of caries. Moreover, as the cells need to be autologous, a majority of the current adult population will not be able to avail the benefits of this technology.

Finally, SCAP are stem cells that are isolated from developing teeth. Therefore, it is highly improbable that they can serve as viable source for stem cells from a clinical perspective.

## Limitations of growth factor delivery systems

Current approaches to pulp tissue engineering and regenerative medicine in general focus on the use of growth factor delivery systems to deliver one or more growth factors to induce lineage specific differentiation of stem cells. Several natural and polymeric growth factor delivery systems have been developed for controlled delivery of growth factors (Vo et al., 2012). However, the timing of delivery, long-term stability and dosage of these growth factors limit the translational potential severely. Another important consideration is that *in vivo*, the stem cells use several different growth factors at different stages of differentiation and in unique combinations to achieve and maintain lineage specific differentiation. Therefore, it is not realistic to expect that a single growth factor or a handful of growth factors can generate the same effect as the complete extracellular environment that the cells perceive *in vivo*.

## Alternative stem cell sources

With the limited availability of dental stem cells, the need for exploring other stem cell sources for clinical translation is high. Published studies have shown that stem cells isolated from the bone marrow, adipose tissue, and placenta have similar epigenetic profiles (Aranda et al., 2009). However, in one study that compared bone marrow derived mesenchymal stem cells (BMSCs) and adipose tissue derived mesenchymal stem cells (ADMSCs) for pulp tissue regeneration in a canine model, BMSCs were not as potent as DPSCs or ADMSCs (Ishizaka et al., 2012). This study required the use of growth factor delivery (SDF1) and a side population of CD31(−) cells. From a translational perspective, the safety and dosage of SDF1 has not been established and to obtain side populations of autologous stem cells is difficult. Therefore, an efficient system that can direct lineage specific differentiation of alternate stem cell sources that does not rely on growth factor delivery is required.

The bone marrow is self-renewing and BMSCs have clinically standardized isolation procedures and are relatively easy to obtain compared to other stem cell sources. Although not ideal for pulp tissue regeneration using conventional approaches, translational feasibility is highest for BMSCs as opposed to dental stem cells. Both DPSCs and BMSCs are of mesenchymal origin. DPSCs are derived from the neural crest and BMSCs from the bone marrow. This fundamental difference in these two stem cell types results in different behavioral characteristics (Shi et al., 2001; Yu et al., 2007). However, if provided with appropriate extracellular environment, our results indicate that BMSCs can undergo odontogenic differentiation.

Another important consideration is the ability of BMSCs to confer an innate ability to defend against infection. Several published reports highlight the roles and importance of BMSCs in modulating the maintenance and migration of hematopoietic stem cells (HSCs) and monocyte emigration (Mendez-Ferrer et al., 2010; Ehninger and Trumpp, 2011; Shi et al., 2011). As a direct role-player in action against infection, BMSCs can actively prolong survival during sepsis, reduce microbial growth, and burden post infection (Nemeth et al., 2009; Krasnodembskaya et al., 2010; Mei et al., 2010; Meisel et al., 2011). Considering these factors, BMSCs form the ideal source for clinically relevant pulp tissue regeneration.

## The extracellular matrix (ECM)

Living organisms are made up of tissues that consist of live cells embedded within an **ECM**. The ECM constitutes framework of structural and functional proteins within which cells reside *in vivo* (Ravindran and George, 2014). The ECM dictates and defines tissue/organ architecture, shape, and functionality. The ECM is unique for each cell type and consists of a combination of structural and functional proteins in well-defined proportions. The exact composition of the ECM of each cell type still remains elusive. Recent proteomic studies have shown that the ECM of is comprised of thousands of proteins ranging from structural proteins and growth factors to even transcription factors (Alves et al., 2011). Several proteins believed to be intracellular in nature are being discovered in the ECM possessing important extracellular roles (Ravindran and George, 2014). We have identified two such proteins namely; glucose regulated protein 78 (GRP78) (Ravindran et al., 2012a) and TGFβ receptor interacting protein 1 (TRIP1) (Ramachandran et al., 2012) that possess extracellular roles apart from their well-characterized intracellular functions.

KEY CONCEPT 3 **Extracellular matrix (ECM)**

The ECM is the cell-secreted environment within which cells are embedded. The ECM and cells together constitute a tissue. Several tissues possess a complex architecture consisting of multiple cell types and their ECM arranged in a defined architecture to coherently perform a function. The three layers of an artery is a simple example. The ECM defines tissue functionality (bone, cartilage etc.) and governs cell behavior, growth, and differentiation.

Considering the nature and complexity of the ECM, present strategies aimed at using a few factors seem inadequate to accomplish and maintain the engineered tissue. Many studies have suggested the use of decellularized tissues as allografts and xenografts (Badylak et al., 2011; Crapo et al., 2011; Sabetkish et al., 2014). However, as the ECM for each cell type is unique, it is not a feasible strategy to use the matrices derived from other decellularized tissues for dental pulp tissue regeneration. On the other hand, decellularized dental pulp tissues can be a viable option if appropriate protocol to obtain them and store them in substantial quantities is available. This can be a daunting task to accomplish. Therefore, the ideal and clinically relevant strategy would be to recreate the dental pulp environment *in vitro* using an approach that can be transitioned into a mass-producible environment.

## Use of biomimetic scaffolds

**Figure 1:**
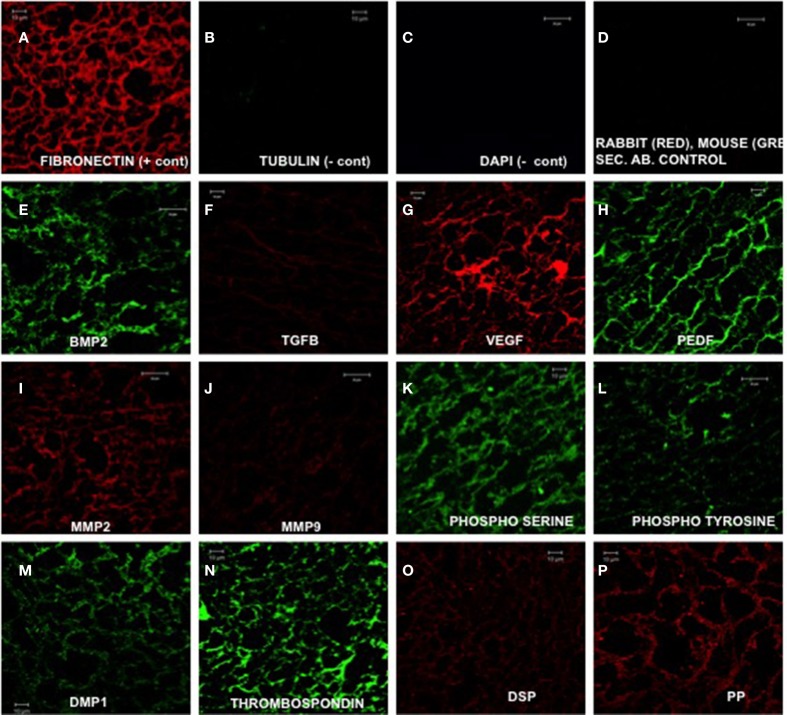
**Expression of ECM proteins (Ravindran et al., 2014b). (A–P)** are representative confocal micrographs showing the expression of various ECM proteins in the ECM scaffold sections. Fibronectin **(A)** was used as a positive control and tubulin **(B)** was used as a negative control for intracellular protein. DAPI staining **(C)** was performed to rule out the presence of DNA material in the ECM scaffold. **(D)** shows absence of rabbit and mouse non-specific secondary antibody binding. **(E–H)** show the presence of growth factors BMP2 (bone morphogenetic protein 2), TGFβ1 (transforming growth factor β1), pro angiogenic factor VEGF, and anti angiogenic factor PEDF (pigment epithelium derived factor), respectively. **(I,J)** show the presence of matrix metalloproteases 2 and 9, respectively. **(K,L)** show the presence of phosphorylated proteins in the ECM by localizing the presence of phosphorylated serines and tyrosines. **(M–P)** show the presence of non-collagenous proteins DMP1 (dentin matrix protein 1), thrombospondin, DSP (dentin sialoprotein), and DPP (dentin phosphophoryn). Scale bar represents 20 μm in **(C–E, I, J, L**). Scale bar represents 10 μm in all other images.

Our approach is focused on creating an environment that mimics the native pulp environment to illicit an appropriate stem cell response and behavior. We have created an ECM based **biomimetic** scaffold that contains an intact ECM of differentiating dental pulp stem cells (Ravindran et al., 2014b). Ours and other published studies have shown the effectiveness of ECM derived scaffolds for inducing lineage specific differentiation of stem cells for several purposes including bone, cartilage, and vascular tissue(Bourget et al., 2012; Ravindran et al., 2012a; Levorson et al., 2014a). However, our approach to pulp tissue engineering is unique in that we aim to differentiate BMSCs to a pulpal lineage using a pulp-specific ECM scaffold. Our studies show that that this scaffold contains both structural and functional proteins required for pulp tissue regeneration (Figure 1) (Ravindran et al., 2014b).

KEY CONCEPT 4 **Biomimetics**

With respect to the field of tissue regeneration, biomimetic refers to strategies that aim to mimic the native biological environment. Tissues in the human bodies are extremely complex entities. Their architecture and composition have evolved over millions of years to achieve specific functions. Therefore, attempting to redesign them is a futile effort. The emerging field of biomimetics aims to recreate the natural environment for use in regenerative medicine. In this article, biomimetic refers to the native cell-generated pulp ECM that is incorporated within the collagen/chitosan framework.

## Advantages of using our native ECM incorporated biomimetic scaffold

Collagen and chitosan constitute the base materials of this biomimetic scaffold. These are both naturally occurring biopolymers that have been shown to be biocompatible and biodegradable without causing adverse immune reactions (reviewed in George and Ravindran, 2010). Additionally, chitosan also possesses antimicrobial properties (Kim et al., 2003) that can potentially prevent secondary infections during and after pulp tissue regeneration especially after the native pulp has been under bacterial attack. In combination with the immunomodulatory property of BMSCs, we can expect to have a potent system to actively fight infection.

The ECM itself is secreted by the cells, consists of structural and functional proteins present in the pulp tissue and is incorporated three dimensionally within the collagen/chitosan framework. One of the salient features of the ECM is that it presents a completely engineered pulp environment containing physiologically relevant amounts of growth factors, cytokines, and metalloproteases. Structural proteins such as collagen and fibronectin can sequester the growth factors and present them to the cells in a manner that mimics the biological scenario *in vivo*.

From a translational perspective, the biomimetic scaffolds can be mass-produced using human pulp cell lines. Published studies have demonstrated the safety of telomerase transfected human cell lines (Nakahara et al., 2009; Bourgine et al., 2014). However, for the generation of ECM incorporated scaffolds, cell lines will only be used to generate the scaffold. Patient specific somatic stem cells (DPSCs if available of BMSCs), can then be used for regeneration. Therefore, there is little chance of oncogenic transformation and a high potential for clinical translation.

## The ECM scaffold can trigger the BMSCs to differentiate toward an odontogenic lineage

**Figure 2:**
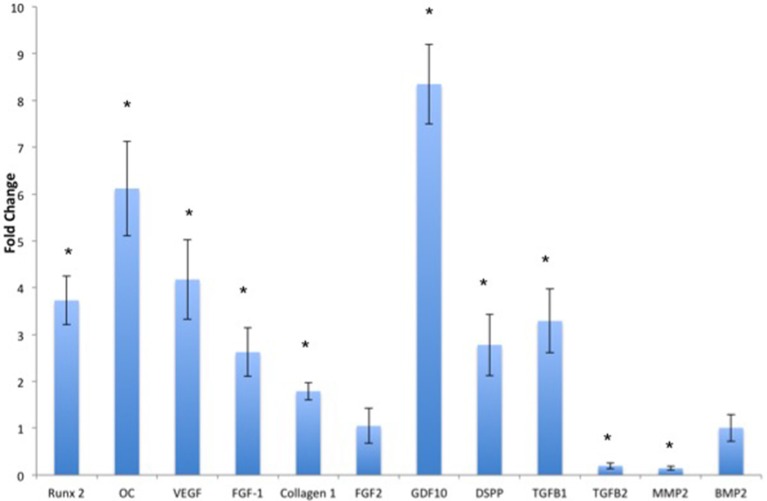
**Quantitative real time PCR analysis**. The bars represent mean fold change in gene expression by the HMSCs cultured within the pulp ECM scaffolds with respect to HMSCs cultured within control collagen/chitosan scaffolds. The experiments were performed in quadruplicates. The error bars represent standard deviation. The statistical significance was calculated using student's *t*-test. ^*^Represents significant change with *P* < 0.05.

The ECM scaffolds can initiate and maintain odontogenic differentiation in DPSCs, BMSCs, and PDLSCs without the need for external delivery of growth factors or differentiating agents (Ravindran et al., 2014a). Of the three cell types, BMSCs are best suited for translation into a clinical setting owing to their ready availability and standardized isolation technique. Figure 2 shows the fold change in gene expression of human marrow stromal cells (HMSCs) cultured within the ECM scaffolds with reference to those cultured within control (non-ECM) collagen/chitosan scaffolds. Our published data shows the behavior of DPSCs and PDLSCs in the same environment (Ravindran et al., 2014a). Our results indicate that the three cell types perform differently in similar environments. However, the end result was a pro-odontogenic differentiation as evidenced by positive regulation of the dentin sialo phospho protein (DSPP) gene. Thus, the gene expression profile shows the potential of the pulp ECM scaffolds to direct odontogenic differentiation of BMSCs as well as DPSCs and PDLSCs.

**Figure 3:**
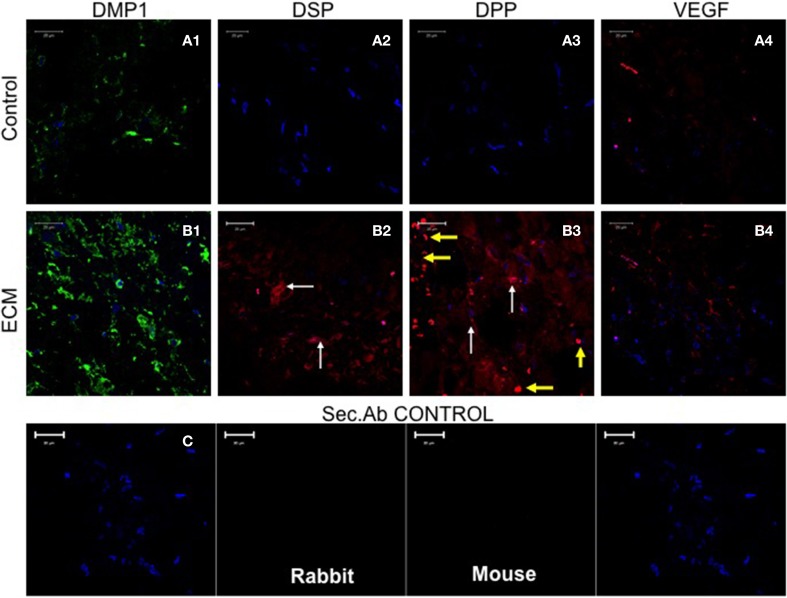
**Fluorescence Immunohistochemistry**. The fluorescent images are representative confocal micrographs of sections from HMSC seeded scaffold explants consisting of control collagen/chitosan scaffold **(A)** and pulp ECM scaffold **(B)**. The sections were analyzed for the expression of DMP1 **(A1,B1)**, DSP **(A2,B2)**, DPP **(A3,B3)**, and VEGF **(A4,B4)**. Note the absence of DSP and DPP signal in **(A)** (control scaffolds containing HMSCs). White arrows in **(B)** point to positive staining of the same by HMSCs seeded within the pulp ECM scaffold. Yellow arrows in **(B3)** point to non-specific fluorescence from red blood corpuscles (RBCs). The presence of RBCs also indicate the presence of active capillaries. Note the increase in DMP1 (comparing images **A1** and **B1**) and VEGF (comparing images **A4** and **B4**) expression. The secondary antibody control for rabbit and mouse secondary antibodies did not show any staining **(C)**. The imaging conditions were maintained constant. The scale bar represents 20 μm in all images.

The potential of the pulp ECM scaffolds were also evaluated *in vivo*. When implanted subcutaneously in immunocompromiced mice, the biomimetic ECM scaffolds triggered odontogenic differentiation of DPSCs, PDLSCs, and BMSCs (Ravindran et al., 2014a). Figure 3 shows odontogenic differentiation of BMSCs *in vivo*. BMSCs expressed the marker proteins DSP and DPP suggesting positive differentiation toward the odontogenic lineage. These experiments showed clearly that the BMSCs possess the potential for odontogenic differentiation if they are provided with an appropriate extracellular environment. Additionally, the experiments also showed that the pulp ECM incorporated scaffolds, can trigger odontogenic differentiation of different types of mesenchymal stem cells and that all the cell types do not follow similar pathways to achieve lineage specific differentiation.

## The biomimetic ECM scaffolds can trigger vascularization *in vivo*

**Figure 4:**
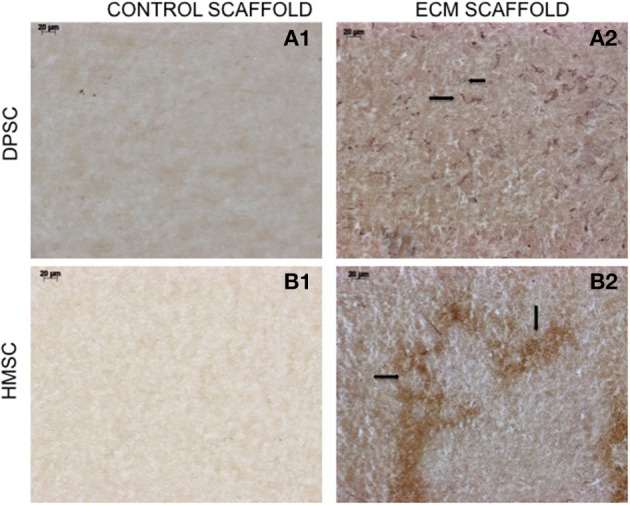
**von Willebrand factor (VWF) immunohistochemistry**. The micrographs are representative images of VWF stained explant sections containing DPSCs **(A1,A2)** and HMSCs **(B1,B2)** in control and pulp ECM scaffolds. The arrows in the images point to positively stained endothelial cells (black arrows). Note the absence of endothelial cells in the control scaffolds containing both DPSCs **(A1)** and HMSCs **(B1)**. The scale bar in all the images represents 20 μm.

The data presented in Figure 1 shows that the ECM scaffolds, amongst other proteins, contains pro-vascular growth factors such as vascular endothelial growth factor (VEGF). When these scaffolds were implanted subcutaneously in nude mice, they showed more robust vascularization compared to control scaffolds indicating their ability to promote vascularization (Figure 4). The data presented in the figure show that the ECM scaffolds promote vascularization in the presence of both DPSCs and BMSCs. Our published results show vascularization in the presence of DPSCs, BMSCs, and PDLSCs (Ravindran et al., 2014a). The images in Figure 4 show the presence of endothelial cells that stained positive for von Willebrand factor. Note the presence of capillary-like structures in the ECM scaffold containing BMSCs.

One of the primary challenges associated with dental pulp tissue engineering is vascularization of the tissue. The apex of the root canal space is small. It is also the only opening through which the tissue can be vascularized. It is imperative that any tissue engineering technology that attempts to regenerate the dental pulp is pro-vascular in nature. Therefore, the ability of the biomimetic ECM scaffolds to induce vascularization will be highly beneficial toward clinical translation of this approach to pulp tissue regeneration.

## Implications of this biomimetic technology on other fields of tissue engineering

In this review, we have discussed the uniqueness of cell type—specific ECM. The generation of biomimetic scaffolds is therefore not limited to dental pulp tissue regeneration. It can be applied toward all fields of tissue engineering. Other groups and us have tested this strategy for regeneration of bone (Thibault et al., 2010; Ravindran et al., 2012a). Recently, similar strategies have been attempted for generating ECM incorporated scaffolds for cartilage tissue regeneration (Levorson et al., 2014a,b). Apart from being used for generating new biomaterials, this strategy can also be used to improve the properties of existing clinical materials. We are presently, working on improving the bioactivity and regenerative capacity of existing clinical materials such as demineralized bone matrices (DBM) using this technology. Additionally, we are also focusing on generation of platforms to aid transplantation surgery (such as biomimetic platforms to enable vascularization for islet transplantation). We envision that in the near future, several medical devices and biomaterials that are currently in use and biologically inert, can be improved using this biomimetic approach.

## Conclusion and future direction

In this focused review, we have discussed the challenges facing clinical translation of dental pulp tissue engineering approaches. We believe that the limited availability of dental stem cells and the limitations of growth factor delivery systems and dosage complications severely hamper the potential of current approaches. Our experiments have demonstrated that it is possible to overcome these hurdles by using biomimetically engineered pulp ECM incorporated scaffolds that we have developed. These scaffolds possess the ability to trigger lineage specific differentiation of several mesenchymal stem cell types *in vitro* and *in vivo*. Therefore, dental stem cells can be avoided if appropriate sources are not available. On the other hand our experiments also show that these scaffolds contain an array of lineage specific proteins that include both structural and functional proteins in physiologically relevant quantities. Therefore, the use of these scaffolds will overcome the need for using complex growth factor delivery systems.

Although the results are promising, several factors need to be standardized before this technology can be ready for clinical translation. To achieve mass-production and minimal batch variability, the ECM incorporated scaffolds need to be produced using dental stem cell lines. Standardized tests need to be developed to assess the quality of the scaffolds and to quantitatively evaluate the levels of different growth factors and ECM proteins within the scaffolds. These tests will ensure product reliability and minimum performance thresholds. Our current research is focused on two objectives: The first objective is to test the effectiveness of these biomimetic scaffolds in a large animal pulpectomy model to evaluate vascularization, innervation, and overall complete regeneration of intact pulp tissue. The second objective that we are working on is to standardize their potential for mass production using dental pulp stem cell lines and bioreactors. We envision that if we are able to accomplish both of these objectives, these biomimetic ECM scaffolds have immense potential to successfully replace root canal therapy with a regenerative therapy that will maintain tooth vitality, functionality, and longevity. Results from these studies will serve as a proof of concept for application to other fields of regenerative medicine.

Overall, our goal is to enhance the translational potential of tissue-engineering strategies by eliminating the need for growth factor delivery systems and sub population of stem cells that have limited availability. This biomimetic technology is a step in this direction.

## Author contributions

SR: First author and corresponding author. Conceptualized, planned, and performed the experiments referenced in this manuscript and wrote the manuscript. AG: Second author. Was involved in conceptualization and planning. Contributed toward editing and proofreading the manuscript.

### Conflict of interest statement

The authors declare that the research was conducted in the absence of any commercial or financial relationships that could be construed as a potential conflict of interest.
